# Prophylactic Use of Troxerutin Can Delay the Development of Diabetic Cognitive Dysfunction and Improve the Expression of Nrf2 in the Hippocampus on STZ Diabetic Rats

**DOI:** 10.1155/2018/8678539

**Published:** 2018-04-12

**Authors:** Songyun Zhang, Lingling Yuan, Lihui Zhang, Caige Li, Jie Li

**Affiliations:** The Second Hospital of Hebei Medical University, No. 215 Heping Road (West), Shijiazhuang City, Hebei Province, China

## Abstract

**Background:**

With the change in lifestyle and the aging population, the incidence of cognitive dysfunction in diabetes mellitus is rising sharply. Oxidative stress is an important mechanism in the development of diabetic cognitive dysfunction. Nuclear factor E2-related factor 2 (Nrf2) is the core transcription factor of antioxidative stress. Early prevention and treatment of diabetic cognitive dysfunction can reduce the incidence of dementia and improve the quality of life of diabetic patients.

**Aim:**

This study was aimed at determining effect of troxerutin on the development of cognitive dysfunction and the expression level of Nrf2 in the hippocampus of streptozotocin (STZ) diabetic rats, when used in the early preventive stage.

**Methods:**

An STZ-induced diabetic rat model was established (*n* = 30), and the animals were randomly divided into 2 groups: diabetic control group (DC, *n* = 15) and diabetic troxerutin intervention group (DT, *n* = 15). Another 10 normoglycemic rats were put into a normal control group (NC, *n* = 10). While the DT group was injected with troxerutin (60 mg/kg), the DC group and the NC group were injected with physiological saline for 12 weeks daily. Learning and memory behaviors were tested using the Morris water maze test. The superoxide dismutase (SOD) activity, malondialdehyde (MDA) content, mRNA level, and protein level of Nrf2 were measured. Data were collected and analyzed by the statistical software package SPSS 19.0, which included one-way analysis of variance with completely randomized design.

**Results:**

Learning and memory levels were significantly improved in the DT group compared with the DC group. Moreover, in the DT group, the expression level of Nrf2 in the hippocampus was increased, activity of SOD was elevated, and MDA content was decreased.

**Conclusion:**

Prophylactic use of troxerutin delays the development of diabetic cognitive dysfunction and increases the expression level of Nrf2 in the hippocampus of STZ diabetic rats.

## 1. Introduction

As diabetes mellitus can cause cognitive dysfunction, it has become an important research domain. However, the exact mechanisms of diabetic encephalopathy are yet to be identified. Nevertheless, increasing evidence indicates that oxidative stress is an important mechanism in the development of diabetic cognitive dysfunction. Malondialdehyde (MDA) content and superoxide dismutase (SOD) activity are classic indicators in assessing the balance between oxidative and antioxidant systems. Nrf2 is considered to be the main transcription factor of antioxidative stress [1, 2]. In addition, Nrf2 is closely related with cognitive dysfunction [3, 4].

Troxerutin is a flavonol (a type of flavonoid) or, more accurately, a hydroxyethylrutoside. Troxerutin has been reported to possess strong antioxidant and anti-inflammatory properties [5–9]. Importantly, early prevention and treatment of diabetic cognitive dysfunction can reduce the incidence of dementia and improve the quality of life of diabetic patients [3–7]. This study aimed to observe the development of cognitive dysfunction and determine the expression levels of Nrf2 in the hippocampus of streptozotocin (STZ) diabetic rats when using troxerutin in the early preventive stage.

## 2. Results

### 2.1. Morris Water Maze Test

#### 2.1.1. Basic Swimming Speed

There was no statistical difference in swimming speed between the three groups (Table 1).

#### 2.1.2. Navigation Test

Repeated measures ANOVA was used to compare the data between each group. The effect of time did not result in significant changes among the groups (*P* = 0.058). The escape latency of the DC group was significantly longer when compared with that of the NC group (*P* < 0.001). This indicated that diabetic rats had cognitive dysfunction after having successfully modeled for 12 weeks. The DT group had a shorter escape latency than the DC group (*P* < 0.001). This suggests that treatment with troxerutin for 12 weeks can reduce cognitive dysfunction. There were no significant differences in escape latency among the NC, DC, and DT groups after the first day (day 1, *P* = 0.256). Compared with the NC group, the escape latency of DC rats was longer during the subsequent 4 days (day 2, *P* < 0.001; day 3, *P* < 0.001; day 4, *P* < 0.001; day 5, *P* < 0.001). On day 2, the escape latency of the DC group was not significantly different from that of the DT group (*P* = 0.061). From the third to the fifth day, the escape latency of the DT group was significantly shorter than that of the DC group (day 3–day 5: *P* < 0.001) (Table 2).

#### 2.1.3. Probe Test

When comparing the number of times the rats crossed to the platform in 60s, the number of the NC group was greater than those of the DC group and DT group. Moreover, the number of the DT group was greater than that of the DC group (*F*_2,27_ = 185.33, *P* < 0.001) (Table 3).

### 2.2. HE Staining

**Figure 1:**
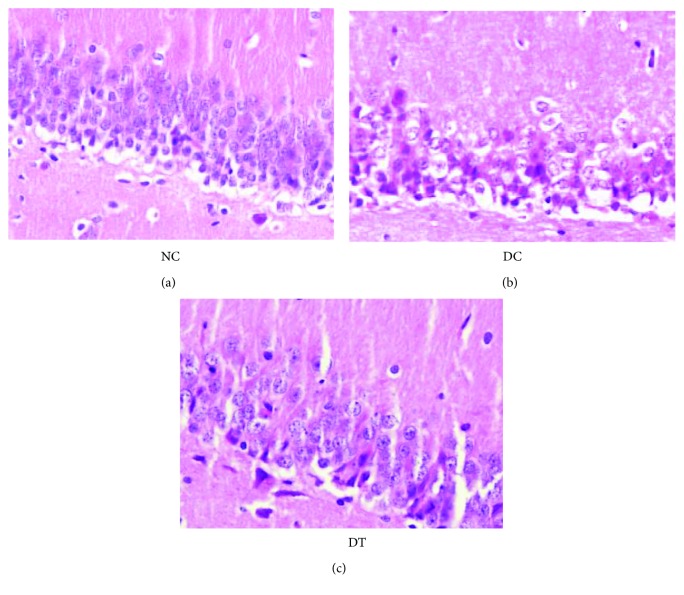
Representative photomicrographs of the hippocampal CA1 area showing the histological changes in different groups (HE, ×200). NC: normal control group; DC: diabetic control group; DT: diabetic troxerutin intervention group.

After observing a series of histopathologic alterations in the hippocampal neurons of the DC group, the number of normal neurons significantly decreased. On the contrary, the majority of the CAl neurons in the DT and NC groups maintained normal morphology, showing integrated structures and being lined up in order. Moreover, there was less neuron depletion in the DT and NC groups compared to the DC group (Figure 1).

### 2.3. Immunohistochemical Staining

**Figure 2:**
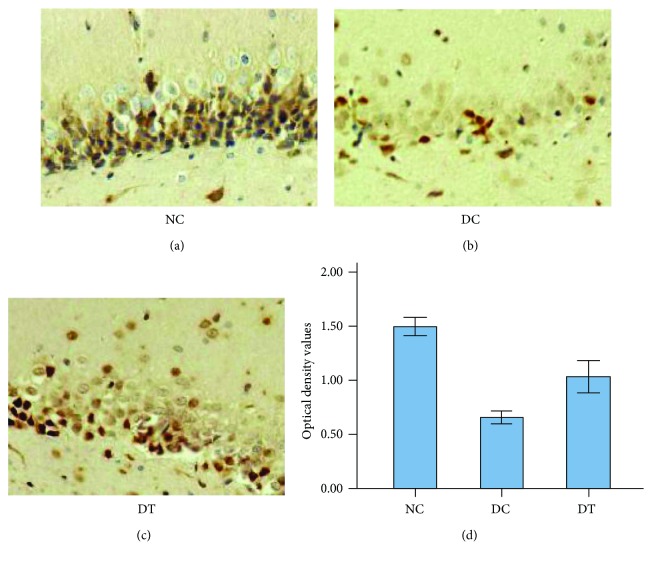
The optical density values of Nrf2 in hippocampal CA1 area of each group (IHC, ×200). Results shown are mean ± SD (*n* = 6). *P* < 0.001 DT versus DC.

The optical density (OD) value of the Nrf2-positive cells in the hippocampus of rats in the NC group was higher than that of rats in the DC group and the DT group. Compared with the DC group, the OD value of the Nrf2-positive hippocampal cells of rats in the DT group was increased (*F*_2,87_ = 64.67, *P* < 0.001) (Figure 2).

### 2.4. Nrf2 mRNA Expression

**Figure 3:**
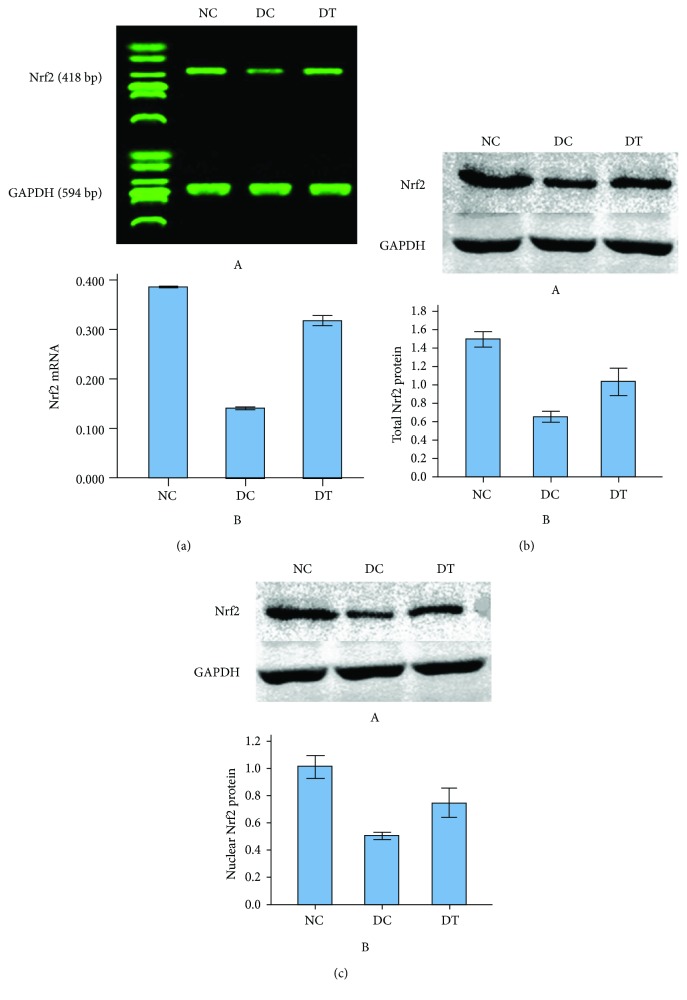
(a) The expression of total Nrf2 mRNA in different groups. Results shown are mean ± SD (*n* = 6), *P* < 0.001 DT versus DC. (b) The expression of total Nrf2 protein in different groups. Results shown are mean ± SD (*n* = 6), *P* < 0.001 DT versus DC. (c) The expression of nuclear Nrf2 protein in different groups. Results shown are mean ± SD (*n* = 6), *P* < 0.001 DT versus DC.

When compared with the NC group, mRNA expression of Nrf2 decreased in the DC group and the DT group. Moreover, there was a statistically significant difference between the DC group and the DT group, the Nrf2 mRNA level being higher in the latter (*F*_2,15_ = 2815.91, *P* < 0.001) (Figure 3(a)).

### 2.5. Nrf2 Protein Expression

Total Nrf2 protein: The expression level of total Nrf2 protein in the hippocampus of rats in the DC and the DT groups was lower than that of rats in the NC group. Moreover, the total Nrf2 protein expression of the DT group was significantly higher than that of the DC group (*F*_2,15_ = 107.95, *P* < 0.001) (Figure 3(b)).

Nuclear Nrf2 protein: The expression level of nuclear Nrf2 protein in the hippocampus of rats in groups DC and DT was lower than that of rats in the NC group. Furthermore, the expression level of nuclear Nrf2 protein of rats in the DT group was higher than that of rats in the DC group (*F*_2,15_ = 65.73, *P* < 0.001) (Figure 3(c)).

### 2.6. SOD Activity and MDA Content

**Figure 4:**
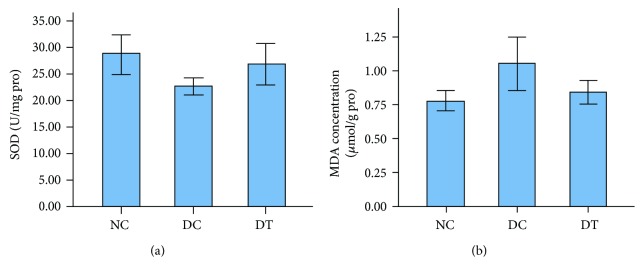
(a) The activity of SOD in different groups. Results shown are mean ± SD (*n* = 6), *P* < 0.05 DT versus DC. (b) The MDA concentration in different groups. Results shown are mean ± SD (*n* = 6), *P* < 0.01 DT versus DC.

SOD activity: The SOD activity of the NC group was higher than that of the DC group. There was no statistical significance between groups NC and DT. However, the SOD activity of the DT group was increased compared with that of the DC group (*F*_2,15_ = 5.873, *P* = 0.013) (Figure 4(a)).

MDA content: The MDA content of the NC group was lower than that of the DC group. There was no statistical significance between the NC group and the DT group. However, the MDA content of the DT group was decreased compared with that of the DC group (*F*_2,15_ = 7.868, *P* = 0.005) (Figure 4(b)).

## 3. Discussion

Modeling is crucial during an experiment, because if an animal model fails, the experimental results will not be reliable.

To successfully develop the rat model of type 1 diabetes mellitus, rats with blood glucose level ≥ 16.7 mmol/L at 72 h after an STZ injection (60 mg/kg) were included in the model.

Rats with blood glucose level < 16.7 mmol/L were rejected once a week [10, 11]. According to the test proposed by Wang et al. [12], 72 SD male rats, aged 8 weeks, were used. Type 1 diabetes mellitus rats began to show cognitive dysfunction symptoms after 6 weeks. More importantly, the symptoms became more apparent after 12 weeks. In this study, rat cognitive function was tested via the Morris water maze, and the results demonstrated that type 1 diabetes mellitus rats became cognitively dysfunctional after 12 weeks.

Troxerutin is 3-hydroxy-rutin and soluble in water [13]. Compared to the intragastric administration, intraperitoneal injection has shorter administration time and is easier to operate. A liquid volume equivalent to 3–8% of animal weight can be absorbed in one hour. This is because a larger area of the peritoneum and clouds of blood vessels are exposed, and the peritoneum has a particularly strong lymphatic absorption capacity for the drug. Clinical trials found that troxerutin has a high safety profile, tolerability, and other advantages, even at high doses [13]. Moreover, a previous study [14] confirmed that 150 mg/kg/d (ig) is the optimal dose for treatment of cognitive dysfunction of diabetic rats.

Furthermore, the dose administered to the rats was equivalent to that previously administered to mice, and the dose administered intraperitoneally was 0.3 to 0.5 times greater than the dose administered intragastrically.

Therefore, in this experiment, rats in the DT group were injected with 60 mg/kg/d (i.e., 150 × 0.4 mg/kg/d) of troxerutin.

In the present study, the Morris water maze test was proven to be a credible test for cognitive dysfunction related to hippocampal synaptic plasticity [15]. The navigation test was used to test the spatial learning ability of rats, whereas the probe test was used to test their spatial memory. The basis of cognitive dysfunction in pathology is the connecting dysfunction of the prefrontal-striatum-thalamus-temporal lobe, which features the hippocampus and plays an important role [16]. The hippocampus is one of the most important nerve centers in learning and memory. Its structure and function are closely linked to diabetic cognitive dysfunction [17]. SOD activity reflects antioxidant levels, and MDA content reflects oxidative levels; thus, these measurements are frequently used to assess oxidative damage [18]. Our results were consistent with those of other recent studies, in that troxerutin has strong antioxidant and anti-inflammatory properties [19]. An important cellular defense mechanism under oxidative stress is the Nrf2-mediated expression of endogenous antioxidants [20].

In this experiment, hippocampus tissues were stained with HE, and CA1 hippocampal nerve cells of rats in the NC group were eumorphic, structurally integral, and ordered; cells in the DC group were disordered, with shrunken cell bodies and red cytoplasm, and the number of nerve cells with normal morphology was significantly reduced; cells in the DT group were arranged in order, structurally integrated. These results suggest that the hippocampal damage in the DC group is morphological, which affects the cognitive function. The degree of hippocampal impairment in the DT group was lower than that in the DC group; thus, the preventive use of troxerutin could alleviate damage to the hippocampus and reduce cognitive impairment, to a certain extent.

In this experiment, the blood glucose level of rats in the DT group was not significantly different from that of rats in the DC group. This illustrates that troxerutin cannot decrease blood glucose level, which agrees with the results of Geetha et al., who tested 36 rats weighing 25~30 g by administrating a high-fat, high-carbohydrate diet. The results suggested that troxerutin reduces body weight, blood pressure, and insulin resistance [21], but does not induce a significant decrease in blood glucose level. In this study, the results of the Morris water maze test proved that the prophylactic use of troxerutin can delay the development of diabetic cognitive dysfunction in STZ diabetic rats. Additionally, the prophylactic use of troxerutin can increase SOD activity in the hippocampus, suggesting that it can improve the antioxidant ability. Both the mRNA and protein expression of Nrf2 were higher in the DT group than in the DC group. This demonstrated that the prophylactic use of troxerutin in rats increases Nrf2 content in the hippocampus. Therefore, troxerutin administered prophylactically delays the development of diabetes-induced cognitive dysfunction in rats by increasing the Nrf2 content in the hippocampus.

## 4. Methods

### 4.1. Animals and Drug Administration

All procedures involving animals were in accordance with the *Guide for the Care and Use of Laboratory Animals*. Forty male Sprague-Dawley rats (age 6–8 weeks, from Beijing Vital River Laboratory Animal Technology Co., certificate number 1100195543) were randomly divided into a normal control group (NC group, *n* = 10 rats) and a diabetic group (*n* = 30 rats). The rats fasted overnight. Then, STZ was dissolved in 0.1 mol/L sodium citrate buffer, pH 4.4, and the 30 rats were intraperitoneally injected with STZ citrate buffer (60 mg/kg/d, Sigma Company, USA). At 72 hours after the injection, the blood glucose level of the rats was determined via their tail veins using a blood glucose meter. Fasted rats with a blood glucose level above 16.7 mmol/L were considered to be diabetic and were randomly divided between a diabetic control group (DC group, *n* = 15) and a diabetic troxerutin intervention group (DT group, *n* = 15).

Rats in the DT group were intraperitoneally injected with troxerutin (60 mg/kg, 1 mL/kg, Jingchun Biochemistry Technology Corporation, Shanghai, China), while those in the DC and NC groups were intraperitoneally injected with physiological saline once daily for 12 weeks. Finally, the blood glucose levels were measured after 12 weeks of troxerutin treatment.

### 4.2. Morris Water Maze Test

The rats were tested in the Morris water maze at the end of the treatment period (12 weeks). Basic swimming speed of the rats was assessed the day prior to the test. Rats were pretested to determine their treadmill running willingness and those rats which refused to run were excluded from the water maze experiments. The maze consisted of a circular pool (150 cm in diameter and 60 cm in height) that contained a hidden platform. The apparatus was used to assess the learning and memory performance of rats. Rats were trained to locate the hidden platform using only distal extra maze cues.

For the navigation test, the tracks of rats were recorded and analyzed by using a camera connected to a video recorder and the EthoVision tracking system (Zhenghua Bio-instruments Co. Ltd., Huaibei, Anhui). Each rat was allowed to search for the platform for 60 seconds, and rest on the platform for 30 seconds once found. Then, the rats were placed back in their respective cages. Rats that failed to find the platform within 60 seconds were manually guided to the platform and placed on the platform for 30 seconds before being returned to their cages. Each rat received four learning trials in the four quadrants (N, S, E, and W) per day for five consecutive days. The times spent searching for the platform and the mounting latency were calculated.

On the sixth day, the rats were given the probe test, in which the platform was removed from the pool. Rats were randomly placed into a quadrant and were allowed to swim freely for 60 s. The number of times the rats swarm across the platform in 60 s was recorded [15].

### 4.3. Biochemical Assessment

On the seventh day, the rats were sacrificed, and their left and right hippocampus was removed promptly. The right side of the hippocampus was used for hematoxylin and eosin (HE) staining and immunohistochemistry. The left side of the hippocampus was transferred to a 4°C ice-cold board for dissection and then used to measure SOD activity and MDA content and to detect the mRNA (reverse transcription polymerase chain reaction, RT-PCR) and protein (Western blot analysis) levels of Nrf2 in both cytoplasm and nucleus.

#### 4.3.1. HE Staining

The syringe containing the sample was kept vertically and still for 10 to 20 minutes, with the needle end pointing downward. The glass slides were cleaned, disinfected with 75% ethanol, and then dried. One droplet of test solution was added to the glass slide and dried at room temperature. The sample was then fixed in methanol solution for 30 minutes. The methanol solution was removed, and the sample was hydrated with distilled water and subjected to conventional HE staining.

#### 4.3.2. Immunohistochemistry for Nrf2 Expression

Sections were dehydrated in xylene and graded alcohol series. The sections were then rinsed for 5 minutes with tap water and incubated for 30 minutes in 0.3% H_2_O_2_ in methanol. After, they were washed for 5 minutes with buffer and incubated for 20 minutes in diluted normal serum from the species in which the secondary antibody was obtained. Excess serum from sections was blotted. Then, the sections were incubated for 30 minutes in primary antiserum diluted with buffer. Slides were subsequently washed for 5 minutes with buffer. Sections were incubated for 30 minutes in diluted biotinylated secondary antibody solution, followed by a 5-minute washing step in buffer. Sections were then incubated for 30 minutes in VECTASTAIN ABC Reagent and washed for another 5 minutes with buffer. Next, sections were incubated in peroxidase substrate solution until the stain developed to the desired intensity. Lastly, sections were rinsed with tap water, counterstained, cleared, and mounted for observation.

#### 4.3.3. Quantitative Assessment of Nrf2 mRNA by RT-PCR

Four rats from each group were randomly selected. Total RNA from the hippocampus was isolated using Trizol (Invitrogen Co.), according to the manufacturer's instructions. Reverse transcription was performed using the DEPC (USA Co.). The expression of the housekeeping gene glyceraldehyde-3-phosphate dehydrogenase (GAPDH) mRNA was used as an internal standard. PCR was performed for 30 cycles in an Eppendorf Mastercycler. Denaturing, annealing, and extension reactions were performed at 94°C for 30 minutes, 60°C for 30 seconds, and 72°C for 1 minute, respectively. Then, the PCR products were separated by 1% agarose gel electrophoresis, stained with GoldView, photographed under ultraviolet illumination, compared with a known standard ladder, and quantified by densitometry using the Bio-1D system. The levels of Nrf2 mRNA were expressed as their respective ratios to GAPDH mRNA.

#### 4.3.4. Western Blotting Analysis for Determining the Nrf2 Protein Levels

Approximately 500–600 mg of tissue homogenate were added to 1 mL of extraction solution. The mixture was then centrifuged at 12,000 rpm for 5 minutes, at 4°C, and the supernatant was collected. An equal volume of sample buffer was added to the supernatant on *χ*SDS. The sample was then boiled for 5 min at 100°C. The same amount of total protein was added to each well, to undergo polyacrylamide gel electrophoresis. The proteins were transferred to a membrane by electric water bath blotting. After the membrane was shaken in a petri dish for 2 hours at room temperature, primary antibody (diluted 1 : 250 in blocking buffer) [Nrf2 (C-20) is an affinity purified rabbit polyclonal antibody raised against a peptide mapping at the C-terminus of Nrf2 of human origin, Santa Cruz Biotechnology] was added and incubated for 2 hours at room temperature. The membrane was subsequently washed with PBS-Tween three times. After washing, labeled horseradish peroxidase (HRP) secondary antibody (diluted 1 : 5000 in blocking buffer) [GAPDH(0411) is a mouse monoclonal antibody raised against recombinant GAPDH of human origin] was added, and the petri dish was shaken for 1 hour at room temperature. After washing the membrane sufficiently with PBS-Tween, the membrane was immersed in DAB color development solution and was let to sit at room temperature for full color development. Then, the membrane was washed with distilled water and immediately transferred to PBS for observation. Lastly, the membrane was photographed and analyzed.

#### 4.3.5. SOD Activity and MDA Content

SOD activity is measured by testing the capacity of pyrogallol to autoxidize. The inhibition of autoxidation of this compound occurs when SOD (Jian Cheng Technology Corporation, China) is present, and the enzymatic activity can be assayed indirectly using a temperature-controlled double-beam spectrophotometer at an absorbance of 420 nm. A 50% inhibition of pyrogallol autoxidation is defined as 1 U SOD. For testing MDA (Jian Cheng Technology Corporation, China) levels, the samples were mixed with 1 mL of 10% trichloroacetic acid and 1 mL of 0.67% thiobarbituric acid and then heated in a boiling water bath for 30 minutes. Malondialdehyde equivalents were detected in both tissue and submitochondrial particles of the rats' brain using a spectrophotometer at an absorbance of 532 nm.

### 4.4. Statistical Analysis

Data were collected and analyzed by the statistical software package SPSS 19.0, which includes one-factor ANOVA with completely randomized design. Repeated measures ANOVA was used to analyze the results of the Morris water maze test. The method of Bonferroni was used to perform pairwise comparisons of the repeatedly measured data from the different measurement times of each treated group, as previously described [22]. As the data passed the normality test (*P* > 0.10), the results were evaluated as the mean ± standard deviation. Values of *P* < 0.05 were considered statistically significant.

## Figures and Tables

**Table 1:** The basic swimming speed of the rats in different groups (cm/s, $\bar{x}+s$).

Group	*n*	The swimming speed
NC	10	30.67 ± 2.58
DC	10	28.32 ± 1.65^△^
DT	10	30.06 ± 2.58^△▲^

Note: compared to NC, ^△^*P* > 0.05; compared to DC, ^▲^*P* > 0.05.

**Table 2:** The escape latencies of the rats in different groups in place navigation test (s, $\bar{x}+s$).

Group	*n*	Day 1	Day 2	Day 3	Day 4	Day 5
NC	10	22.08 ± 12.22	11.01 ± 4.19	7.82 ± 3.84	7.52 ± 3.06	5.81 ± 2.33
DC	10	29.22 ± 14.10^△^	32.56 ± 15.66^△^	28.58 ± 12.21^△^	28.72 ± 3.59^**△**^	24.68 ± 8.02^△^
DT	10	25.94 ± 15.03^△▲^	25.38 ± 16.21^△▲^	16.00 ± 8.33^△▲^	14.72 ± 6.29^△▲^	15.04 ± 10.20^△▲^

Note: compared to NC, ^△^*P* < 0.01; compared to DC, ^▲^*P* < 0.01.

**Table 3:** The times of crossing platform in 60s in different groups in spatial probe test ($\bar{x}+s$).

Group	*n*	Times of cross the platform area
NC	10	16.00 ± 1.83
DC	10	3.00 ± 0.91^△^
DT	10	13.00 ± 1.83^△▲^

Note: compared to NC, ^△^*P* < 0.001; compared to DC, ^▲^*P* < 0.001.

## Additional Points

*Highlights*. (1) This research emphasizes the importance of preventive intervention. (2) The mechanism of diabetic cognitive dysfunction relies on oxidative stress. (3) The prophylactic use of troxerutin can delay the development of diabetic cognitive dysfunction. (4) The prophylactic use of troxerutin can improve the expression of Nrf2 in the hippocampus of STZ diabetic rats.
